# Morgagni Hernia: Management in a 77-Year-Old Female With a Robotic Approach

**DOI:** 10.7759/cureus.81531

**Published:** 2025-03-31

**Authors:** Abby Kunitsky, Amer Mansoor, Tyler Sauerbeck, David Lang, Raimundo Pastor

**Affiliations:** 1 Internal Medicine, McLaren Macomb, Mt. Clemens, USA; 2 General Surgery, Garden City Hospital, Garden City, USA; 3 General Surgery, Corewell Health - Farmington Hills, Farmington Hills, USA; 4 Internal Medicine, Corewell Health - Farmington Hills, Farmington Hills, USA

**Keywords:** adult morgagni hernia, diaphragmatic hernias, hernia repair, morgagni hernia, robot-assisted, robotic hernia, symptomatic hernia

## Abstract

Morgagni hernias are rare congenital diaphragmatic defects. While often asymptomatic, they can present with nonspecific abdominal or respiratory symptoms, leading to incidental diagnosis through imaging. Surgical repair is the standard treatment to prevent complications such as bowel incarceration or strangulation, though the optimal approach remains a matter of debate. We present the case of a 77-year-old female who was found to have a Morgagni hernia involving the transverse colon. She underwent successful robotic-assisted repair with plication, approximation, and mesh placement. This report highlights the feasibility and safety of robotic-assisted repair for a Morgagni hernia, emphasizing its role in optimizing patient outcomes. As surgical techniques continue to evolve, further studies comparing long-term outcomes of robotic, laparoscopic, and open approaches will help refine best practices for managing this rare congenital defect.

## Introduction

Morgagni hernias are extremely rare in adulthood, estimated to make up 2-4% of all congenital diaphragmatic hernias [1,2]. Development of such hernias occurs secondary to fusion failure between the anterior pleuroperitoneal membrane, sternum, and costal cartilage, resulting in an anteromedial defect in the costosternal trigone known as the foramen of Morgagni [3]. Visceral organs can herniate through the defect, thus resulting in what is known as a Morgagni hernia. A Morgagni hernia defect occurs through the sternocostal triangle, which is also known as the triangle of Larrey. The boundaries of this triangle consist of the sternum anteriorly, the costal cartilage laterally, and the muscular fibers of the diaphragm posteriorly. Other names for this triangle are the foramina of Morgagni, Larrey’s space, and trigonum sternocostale [4]. While these patients are often asymptomatic, they can present with nonspecific abdominal or respiratory symptoms, with subsequent imaging leading to the discovery of the hernia [5].

While any organ can herniate through the defect, the two most common are herniation of the colon or omentum as per the literature [6]. Workup on presentation typically involves CT imaging, preferably of the chest, which demonstrates the presence or portion of visceral organs in the space of the thoracic cavity [7]. The only established treatment for Morgagni hernias in adulthood is surgical repair for both symptomatic and asymptomatic patients; many surgical approaches have been described, but there is no consensus regarding a standard surgical approach, given its rare occurrence and discovery in adult patients [8]. In this report, we discuss our experience with a patient who presented to the emergency department with complaints of epigastric abdominal pain and was found to have a Morgagni hernia involving the transverse colon on CT imaging. She was managed with primary surgical repair using a robotic approach.

## Case presentation

**Figure 1:**
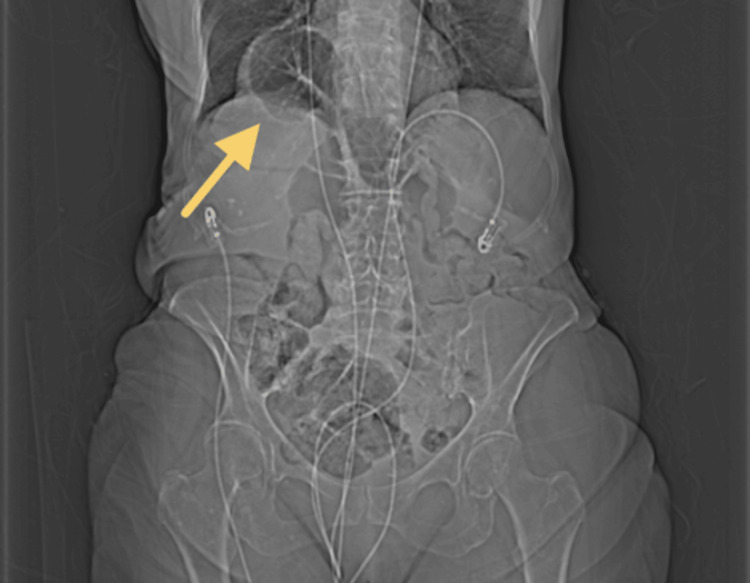
Abdominal X-ray demonstrating Morgagni hernia findings The arrow indicates a short loop of transverse colon herniating through a diaphragmatic defect into the thoracic cavity

**Figure 2:**
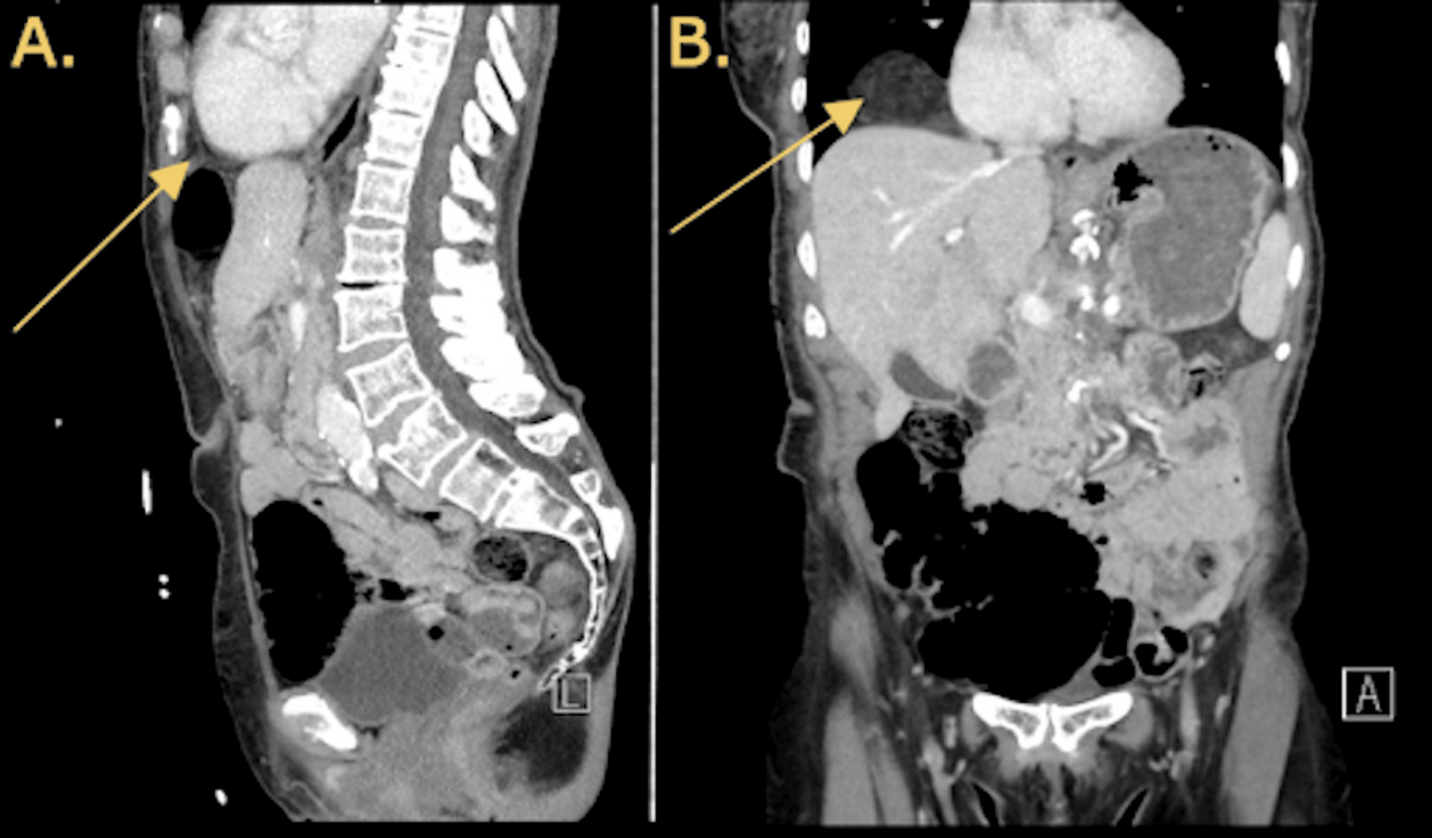
CT imaging of the abdomen and pelvis demonstrating Morgagni hernia findings A) Sagittal CT findings with the arrow indicating a right anterior diaphragmatic defect with a nondilated loop of transverse colon herniating into the thoracic cavity. B) Coronal CT findings with the arrow indicating a right anterior diaphragmatic defect with a nondilated loop of transverse colon herniating into the thoracic cavity CT: computed tomography

A 77-year-old female with a past medical history of neuropathy, multiple sclerosis, and osteoporosis presented to the emergency department with a chief complaint of epigastric abdominal pain. The patient reported an acute onset of her abdominal pain the evening before presentation, which had been associated with nausea. She endorsed a chronic history of episodic epigastric pain; however, she could not recall any inciting events or modifying factors to the pain. She underwent a right upper quadrant ultrasound, which was notable only for cholelithiasis. A spot X-ray and CT abdomen/pelvis demonstrated a right anterior diaphragmatic defect with herniation of a loop of nondilated transverse colon herniating into the thoracic cavity (Figures 1, 2). The CT report revealed no evidence of bowel compromise, perforation, or proximal dilation. However, there was a low clinical suspicion of bowel strangulation. The patient was hemodynamically stable and afebrile with no evidence of leukocytosis or lactic acidosis. There were no notable findings on complete blood count or complete metabolic panel.

**Figure 3:**
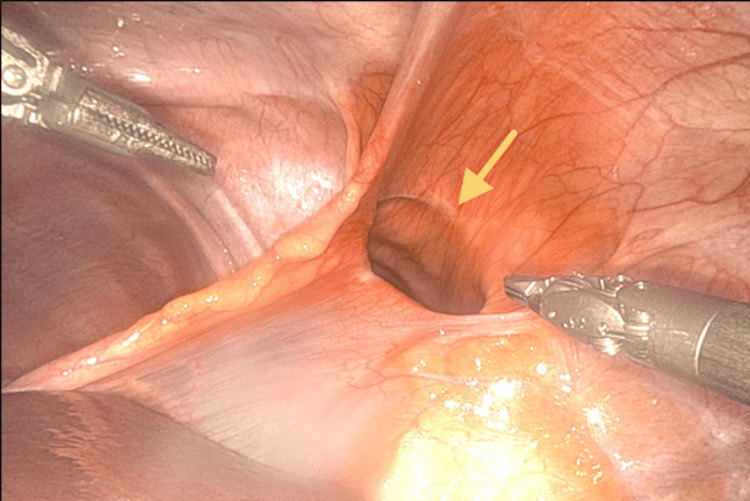
Robotic intraoperative view of the hernia defect The arrow indicates a right anterior diaphragmatic hernia defect from a robotic intraperitoneal view

The patient underwent robotic diaphragmatic hernia repair. This procedure involved obtaining untraditional access with a Veress needle, establishing pneumoperitoneum, and exchanging the Veress needle for an 8 mm robotic port. A 30-degree robotic camera was introduced in the abdominal cavity, with visualization of the anterior diaphragmatic hernia consistent with a Morgagni hernia involving the transverse colon. Two additional 8 mm robotic ports were introduced to the left and right of the rectus complexes. The transverse colon spontaneously reduced from the hernia defect after placing the patient in Trendelenburg, providing good visualization of the hernia defect which measured 3 cm in diameter (Figure 3).

The falciform ligament was divided with monopolar electrocautery scissors. The hernia sac was plicated with a running 2-0 V lock suture. Attempts were made to approximate the diaphragm transversely with a running 0 V lock suture. A 9 cm Symbotex round mesh was then placed in an intraperitoneal onlay position centered under the hernia. The mesh was circumferentially sutured to the anterior abdominal wall, taking care to avoid injury to the pericardium using a 2-0 V lock suture. The incision sites were closed and the case was completed. 

Postoperatively, the patient recovered well and was tolerating a regular diet. A postoperative chest X-ray was performed, which reported no evidence of pneumothorax. She denied any difficulty breathing and had no notable changes in her oxygen saturation or other vitals. She reported that her epigastric pain had subsided and stated that she felt significantly improved overall. The patient tolerated appropriate activity and had no notable findings on her postoperative lab work. She was determined to be stable for discharge on postoperative day one with outpatient follow-up.

## Discussion

The formation of Morgagni hernias occurs due to the failed fusion between the diaphragm and costal arches during embryogenesis [9]. Congenital diaphragmatic hernias in adults can be anterior or posterior. While the anterior hernias on the right and left are known as Morgagni and Larrey hernias, respectively, posterior hernias are known as Bochdalek hernias [10]. Although congenital in nature, previous literature has described an association between the development of Morgagni hernias and prolonged/sudden increase in intra-abdominal pressure during adulthood [9]. Thus, conditions such as chronic constipation, pregnancy, obesity, trauma, and chronic cough can all increase the risk of developing a Morgagni hernia if a patient is predisposed to its development secondary to failed diaphragmatic fusion [11].

Previous systematic reviews of the condition have demonstrated a higher prevalence among females when compared to males (a female-to-male ratio of 2:1) and in patients after their fifth or sixth decade of life [2]. Our patient was a female in her late 70s, which aligns with the findings of those reviews. While symptoms are generally nonspecific, as they were in this case, they often prompt some form of abdominal or chest imaging, which will often lead to a definitive diagnosis [12]. In this case, the patient was evaluated with a right upper quadrant ultrasound, which was largely unremarkable; a subsequent CT of the abdomen and pelvis revealed the hernia defect. However, even though most patients are diagnosed preoperatively, previous reviews have shown that close to a quarter of patients are diagnosed in the intraoperative setting [2].

The gold standard treatment for Morgagni hernia in adults involves surgical defect repair [1,13,14]. The inability to tolerate oral intake, nausea, and vomiting are all symptoms that can raise suspicion for strangulation or incarceration and increase the likelihood of possible ischemia and perforation, making an open approach preferable in these cases [2,5,15]. The approach in elective cases, on the other hand, is often a matter of debate, involving open, laparoscopic, or robotic options through the abdomen or thorax, as mentioned extensively in previously published case reports [16]. Along with the surgical approach, the type of repair is another debated topic with the utilization of either mesh or simple closure of the defect as options, with each being used approximately half of the time [2,6,13].

The operative technique depends on the specific anatomical details and the surgeon’s preference. Researchers have supported the use of a sleeve of composite mesh used as a bolster separate from the "main mesh" [17]. The main mesh used is often a nonabsorbable, permanent mesh such as polypropylene used in a dual layer depending on the defect size and tissue quality. In our case, a mixed approach was utilized with plication of the hernia sac, approximation of the diaphragm transversely, followed by mesh placement. Postoperative chest X-rays are performed at the discretion of the surgeon in abdominal approaches [18]. In our case, given the proximity and primary diaphragmatic repair, a decision to obtain a postoperative chest X-ray was made, which showed no findings that raised concerns for pneumothorax. Chest tube placement is the standard of care in cases approached through the thorax [19].

Despite their rarity, Morgagni hernias require careful consideration in the acute setting due to the potential for complications like strangulation or incarceration, which may necessitate urgent surgical intervention. In our case, the decision to opt for a robotic approach was driven by its advantages in providing precise dissection and repair in a minimally invasive manner, particularly beneficial in elderly patients to reduce postoperative morbidity and facilitate quicker recovery [11]. Robotic surgery offers enhanced visualization and dexterity, making complex anatomical repairs like hernia sac plication and mesh placement, as performed in our patient, more suitable for the surgeons [17].

## Conclusions

Morgagni hernias, though rare, require timely surgical intervention to prevent complications such as incarceration or strangulation. While various surgical approaches exist, robotic-assisted repair offers several advantages, making it a viable option, particularly in elderly patients. This report demonstrates the feasibility and safety of the robotic approach to repairing an adult Morgagni hernia, with successful repair and an uneventful postoperative course. As robotic surgery continues to evolve, further studies comparing long-term outcomes between robotic, laparoscopic, or open approaches, as well as peritoneal, preperitoneal, or thoracic approaches, can help further refine best-practice techniques for the management of this type of congenital diaphragmatic defects.
